# Vps35-deficiency impairs SLC4A11 trafficking and promotes corneal dystrophy

**DOI:** 10.1371/journal.pone.0184906

**Published:** 2017-09-21

**Authors:** Wei Liu, Fu-Lei Tang, Sen Lin, Kai Zhao, Lin Mei, Jian Ye, Wen-Cheng Xiong

**Affiliations:** 1 Department of Neuroscience and Regenerative Medicine, Medical College of Georgia, Augusta University, Augusta, Georgia, United States of America; 2 Department of Neurology, Medical College of Georgia, Augusta University, Augusta, Georgia, United States of America; 3 Department of Ophthalmology, Institute of Surgery Research, Daping Hospital, Third Military Medical University, Chongqing, China; 4 Charlie Norwood VA Medical Center, Augusta, Georgia, United States of America; University of Florida, UNITED STATES

## Abstract

Vps35 (vacuolar protein sorting 35) is a major component of retromer that selectively promotes endosome-to-Golgi retrieval of transmembrane proteins. Dysfunction of retromer is a risk factor for the pathogenesis of Parkinson’s disease (PD) and Alzheimer’s disease (AD). However, Vps35/retromer’s function in the eye or the contribution of Vps35-deficiency to eye degenerative disorders remains to be explored. Here we provide evidence for a critical role of Vps35 in mouse corneal dystrophy. Vps35 is expressed in mouse and human cornea. Mouse cornea from Vps35 heterozygotes (Vps35^+/-^) show features of dystrophy, such as loss of both endothelial and epithelial cell densities, disorganizations of endothelial, stroma, and epithelial cells, excrescences in the Descemet membrane, and corneal edema. Additionally, corneal epithelial cell proliferation was reduced in Vps35-deficient mice. Intriguingly, cell surface targeting of SLC4A11, a membrane transport protein (OH^-^ /H^+^ /NH_3_ /H_2_O) of corneal endothelium, whose mutations have been identified in patients with corneal dystrophy, was impaired in Vps35-deficient cells and cornea. Taken together, these results suggest that SLC4A11 appears to be a Vps35/retromer cargo, and Vps35-regulation of SLC4A11 trafficking may underlie Vps35/retromer regulation of corneal dystrophy.

## Introduction

Retromer that contains two sub-protein complexes-the cargo-selective complex and membrane deformation complex is essential for selective retrieval of transmembrane proteins/cargos from endosomes to trans-Golgi network [1–5]. Vps35 (vaculor protein sorting 35) is the key component of the cargo-selective complex, a trimer of Vps proteins Vps35, Vps29, and Vps26 [2, 3, 6, 7]. Dysfunction of Vps35/retromer is believed to be a risk factor for neuro-degenerative disorders [8], including Parkinson’s disease (PD) and Alzheimer’s disease (AD) for the following reasons. Mutations in Vps35 gene has been identified in patients of late-onset PD [9, 10] and early onset AD [11]. The retromer complex (e.g., Vps35 and Vps26) is decreased in the postmortem hippocampus of AD patients [12]. Vps35 or Vps26 deficient animals display partial AD and PD-relevant neuropathologic deficits, including increased β-amyloid (Aβ) in the hippocampus [12, 13], (a major culprit of AD), and elevated α-synuclein with reduced dopamine neurons in the substantia nigra [14–20] (both PD-linked neuropathologic deficits). Vps35 haploinsufficiency in Tg2576 mouse model of AD enhances Aβ-associated neuropathology [13]. Suppression of Vps35 expression in embryonic hippocampal neurons by in utero electroporation of miRNA-Vps35 results in “degenerative-like” phenotypes [21]. Interestingly, Vps35-deficient mice also showed retinal degenerative pathology. Vps35 is selectively expressed in retinal ganglion cells (RGC) in the retina and the RGC dendrites and axon fibers showed degenerative-like deficits in Vps35 mutant retina [22]. Together, these observations have pointed to a role of Vps35/retromer in preventing neuro-degeneration, supporting the view for Vps35/retromer-deficiency as a general risk factor for neurodegenerative disorders.

Does Vps35-deficiency contribute to the pathogenesis of other tissue/cell degenerative disorders? In light of the following observations, we speculate that Vps35 also plays an important role in preventing non-neuronal cell “degeneration”. Vps35 is widely expressed in numerous cell types, including epithelial cells, and endothelial cells, in addition to neurons [23, 24]. Vps35 is necessary for functions in various cell types, including osteoclasts [25], osteoblasts [26], intestine [24], and neurons [21, 27]…

In our studies of Vps35’s function in the retina, we found that Vps35 is not only expressed in RGCs, but also in cornea. We thus further investigated Vps35’s function in cornea. Cornea is a critical “skin” for eye protection, and a main “ocular media” of the eye that transmit light and provide 70–75% of refractive power [28]. Cornea consists of a stratified non-keratinizing epithelial cell layer, a thick highly aligned collagenous stroma interspersed with keratocytes, and a single cell layered endothelium [29]. Dysregulation of corneal development results in multiple corneal dystrophy syndromes or disorders, such as FECD (Fuchs endothelial corneal dystrophy), CHED2 (congenital hereditary endothelial dystrophy), and CDPD (corneal dystrophy and perceptive deafness) [28–34]. These corneal dystrophy disorders have common features of corneal pathology- loss of cell density, excrescences in the Descemet membrane, and corneal edema [31, 32]. Genetic mutations have been identified in multiple genes in patients with these corneal dystrophy disorders. It is of particular interest to note that mutations in SLC4A11 gene cause corneal disorders including CHED2 and FECD [35–40]. SLC4A11, a member of the SLC4 family, is an integral membrane protein and abundantly expressed in cornea. SLC4A11 has been reported to mediate a variety of functions, including Na^+^ coupled OH^−^ transport, Na^+^ independent H^+^(OH^−^) transport, H^+^/NH3 co-transport, NH3 transport, and water transport [41], and its cell surface distribution is critical for its function [40, 42, 43]. Thus, investigating how SLC4A11 trafficking is regulated is of considerably interest.

In this paper, we provide evidence for loss of Vps35 in mouse cornea causes corneal dystrophy. Vps35 is expressed in developing mouse cornea. Cornea from Vps35 heterozygotes (Vps35^+/-^) mice show morphological features of dystrophy, such as edema-relevant increased cell size and cornea thickness, disorganized cell distribution, altered cell density, and excrescences in the Descemet membrane. Mechanical studies suggest that Vps35 is necessary to promote cell surface targeting of SLC4A11, a membrane transport protein of corneal endothelium. Taken together, these results led to the hypothesis that Vps35 is necessary to regulate SLC4A11’s cell surface distribution and function, which may underlie Vps35 regulation of cornea dystrophy.

## Materials and methods

### Animals and reagents

Vps35 mutant mice have been described previously [13, 21, 25]. Mice were maintained on a standard rodent diet and in a standard facility at Augusta University. Animal care was approved by the Institute of Animal Care and Use Committee (IACUC) at the Augusta University according to the National Institute of Health guidelines.

The following reagents were used: mouse anti-Neuronal Class III ß-Tubulin (Thermo Fisher Scientific, Catalog #:32–2600); rabbit anti-Ki67 antibodies (Abcam, Catalog #: ab15580); chicken anti- ß-galactosidase antibody antibodies (Abcam, Catalog #: ab9361) rabbit anti-SLC4A11 antibodies (Thermo Fisher Scientific, Catalog #: PA5-53730). Rabbit polyclonal anti-Vps35 antibody was generated against the murine Vps35 C-terminal sequence by Cocalico Biologicals, Inc. (PA, USA) as described previously [13]. Phalloidin-fluorescent conjugates were purchased from Molecular Probes (Thermo Fisher Scientific, Catalog #:A12379). For immunofluorescence analysis, the secondary antibodies were Alexa Fluor-488 or Alexa Fluro-594 conjugated anti-mouse or anti-rabbit antibodies (Invitrogen). For Western analysis, the secondary antibodies used were horseradish peroxidase (HRP)-conjugated anti-mouse IgG or anti-rabbit IgG antibodies (Santa Cruz Biotechnology, Inc.). All cell culture reagents were purchased from Thermo fisher.

### Ethics statement

The animal study was performed in strict accordance with the recommendations in the Guide for the Care and Use of Laboratory Animals of the National Institute of Health. The protocol was approved by the Institute of Animal Care and Use Committee (IACUC) at the Augusta University according to the National Institute of Health guidelines (Permit Number: 0000284108).

### Corneal, retina, and lens samples

Vps35^+/+^ or Vps35^+/-^ mice were anaesthetized by CO_2_ and sacrificed by dislocation. Their eyes were excised. For cross sectioning, the eyes were immediately embedded in OCT medium (Sakura Finetek, Torrance, CA), and frozen at -80°C for 2h. 20μm-thick sections were cut on a cryostat and mounted onto SuperFrost Plus slides (Thermo Fisher). For flat-mounted samples, after eyes excised, the corneas were removed and fixed in 4% paraformaldehyde in 0.1 M PBS at 4°C for 24 h. For immunofluorescence staining, the cross-section samples were post-fixed in 4% paraformaldehyde in 0.1 M PBS at 4°C for 4 h.

### H&E staining

For H&E staining, the sections were post-fixed in 4% paraformaldehyde in 0.1 M PBS at 4°C for 10 minutes, washed with distilled water, stained in hematoxylin solution for 1 minute, washed in running tap water for 2 minutes, differentiated in 1% acid alcohol for 5 seconds, washed with running tap water for 1 minute, blued in 0.2% ammonia water for 5 seconds, washed in running tap water for 1 minutes, counterstained in eosin Y solution for 30 seconds, washed with running tap water for 1 minute, then dehydrated with alcohol, cleared with xylene and mounted.

### Immunofluorescence staining and confocal imaging

For immunofluorescence staining analysis, cross-sections were post-fixed with 4% PFA at room temperature for 4h, flat mounts or cross-sections were permeabilized with 0.3% Triton X-100 at room temperature for 20 min, and blocked with 10% horse serum and 5% BSA at room temperature for 2h. Flat mounts or sections were incubated for 24h at 4°C in primary antibodies. Antibodies used were rabbit polyclonal anti-Vps35 (1:1,000), rabbit polyclonal anti-ß-galactosidase (1:1,000), rabbit polyclonal anti-SLC4A11 (1:500), mouse monoclonal anti-α-tubulin (1:1,000), chicken polyclonal anti-ß-galactosidase (1:1,000), phalloidin (1:1,000), and anti-Ki67 (1:1,000). Flat mounts or sections were incubated with appropriate fluorescent secondary antibodies at room temperature for 2h, then incubated with Topro3 (1:5,000) at room temperature for 10min. Slides were mounted using VECTASHIELD mounting medium. The stained slices were imaged using a confocal laser-scanning microscope (Nikon C1 confocal system). The acquired images were processed using the NIS (Nikon) or Adobe Photoshop (Adobe Systems) and analyzed with the ImageJ (http://rsbweb.nih.gov/ij/).

### Western blotting

Cornea tissues were washed twice with ice-cold 1× PBS and resuspended in ice-cold lysis buffer. The lysis buffer comprised 50 mM Tris-HCl pH 7.4, 100 mM NaCl, 10% glycerol, 1% Triton X-100, and was supplemented with proteinase inhibitors cocktail tablets (Thermo Fisher). The lysates were centrifuged at 14,000g for 15 minutes at 4°C. Protein concentrations of the lysates were determined with BCA protein assay kit (Thermo Fisher). Western blotting using ECL and Odyssey infrared imaging system (LI-COR Biosciences) were performed as described previously [19, 20].

### Plasmids, HEK293 Cell culture, and transfection

Wild type (WT) SLC4A11 was amplified from mouse kidney cDNA and cloned into the pEGFP-N1 expression vector. The complete cDNA sequence was verified by DNA sequencing.

Human embryonic kidney 293 (HEK 293) cells were grown in DMEM media (Corning), supplemented with 10% heat-inactivated fetal bovine serum and 0.5% penicillin streptomycin solution, at 37°C. HEK 293 cells were transiently transfected with 2.5 μg of wild-type or mutant SLC4A11 plasmids using polyethylenimine (PEI) (Sigma) as described previously [13, 19]. Cells were seeded at a density of 5 × 10^4^ cells on coverslips for microscopy.

### Statistical analysis

All data were expressed as means ± SEM. Three to four mice per genotype per assay were used. Three to four positions were quantified for immunostaining analyses. Statistical analysis was performed using GraphPad Prism 6 software (GraphPad software, La Jolla, CA, USA). Data were analyzed by unpaired t test for comparison between two groups; or by multifactorial analysis of variance (ANOVA) followed by Tukey's post hoc tests for multiple comparisons with two-tailed test. The significance level was set at P < 0.05.

## Results

### Corneal dystrophy-like deficit with normal lens and retina lamination in Vps35-deficient mice

**Fig 1:**
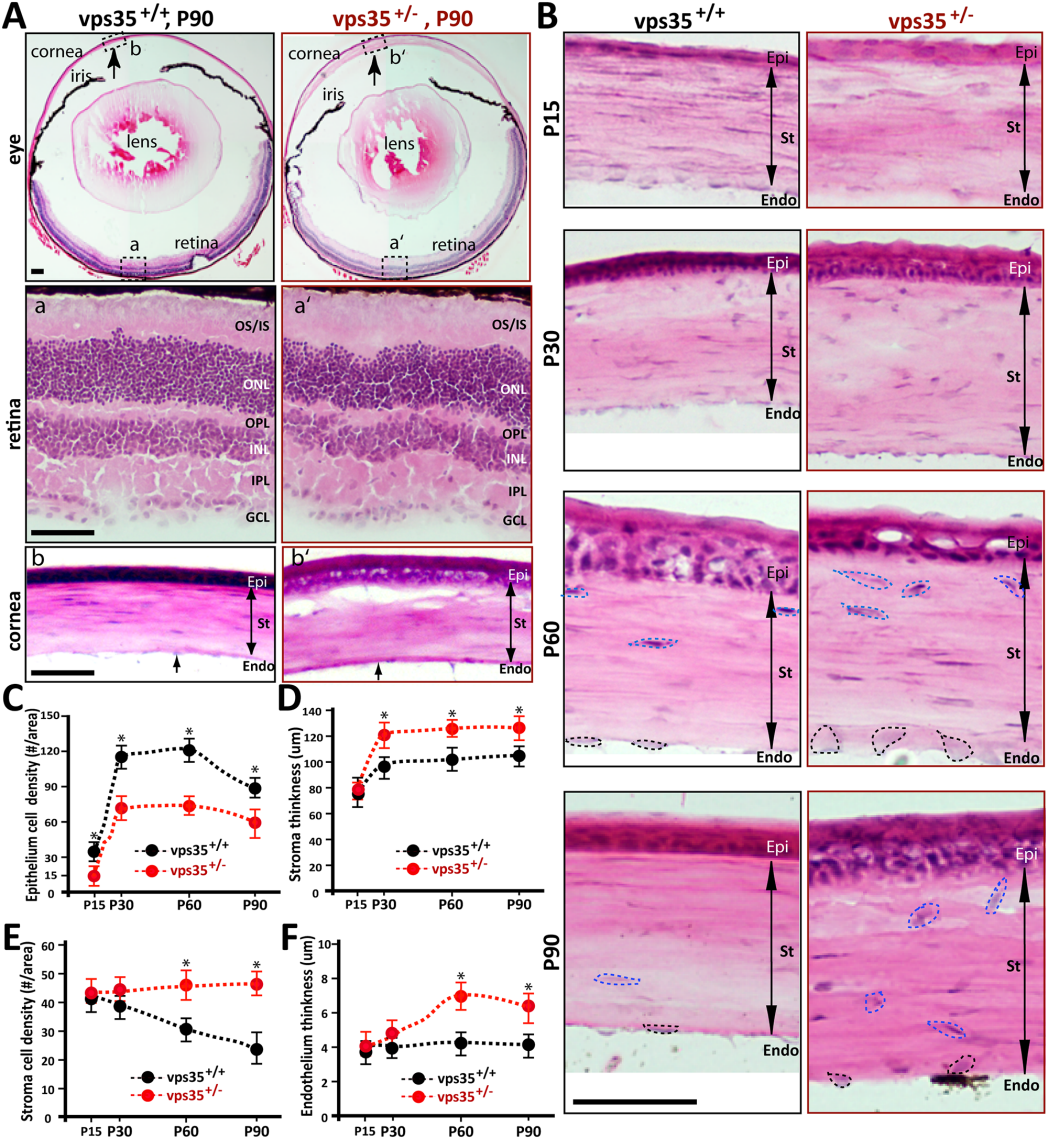
Abnormality of corneal morphology in Vps35^+/-^ cornea. **(A)** H & E staining analysis of cross sections of retina and cornea from P90 old Vps35^+/+^ and Vps35^+/-^ mice. a and a’: retina from Vps35^+/+^ and ^+/-^ mice, respectively; b and b’: cornea from Vps35^+/+^ and ^+/-^ mice, respectively. **(B)** Higher power magnification of H & E staining analysis of cornea from Vps35^+/+^ and ^+/-^ mice at indicated ages. Cells’ nuclei in stroma and endothelium were marked with dotted lines. **(C–F)** Quantification analyses of data from **(B)**. Epithelium cell density was decreased in Vps35 ^+/-^ cornea **(C)**; Endothelium thickness **(F)**, stroma thickness **(D)**, and stroma cell density **(E)** were all increased in Vps35 ^+/-^ cornea. GCL, ganglion cell layer; IPL, inner plexiform layer; INL, inner nuclear layer; OPL, outer plexiform layer; ONL, outer nuclear layer; OS, outer segment; IS, inner segment. Epi, corneal epithelium; St, corneal stroma; Endo, corneal endothelium. Scale bars, 100 μm.

To address whether Vps35-deficiency is a risk factor for retinal neuro-degeneration, we examined the retina morphology in young adult heterozygote (Vps35^+/-^) mice, which have a normal life-span, whereas the homozygotes (Vps35^-/-^) die early during embryonic development [22]. H & E histological examinations of cross sections of whole eye showed a well-organized layer structure without obvious photoreceptor neuron degeneration in Vps35^+/-^ mice, compared to Vps35^+/+^ (WT) controls (Fig 1A). The lens in the mutant eye, viewed by H & E staining and immunostaining analysis of β-catenin, appeared to be normal (Fig 1A and S1 Fig). However, the cornea was obviously thicker and abnormal in Vps35^+/-^ eye (Fig 1A).

We next examined corneal morphology from various ages of WT and mutant mice with higher power magnification (Fig 1B–1F). WT Cornea consists of three layers: a stratified non-keratinizing epithelial cell layer, a highly aligned collagenous stroma, and an endothelial cell layer (Fig 1B). In developing WT epithelium, a remarkable level of differentiation during aging was detected: from one layer of cells in P15 to four or five layers in P60, then reduced to two to three layers of cells in P90 (Fig 1B and 1C). In contrast from WT controls, the mutant epithelial cells from various age-groups were disorganized (Fig 1B and 1C); the thickness of mutant epithelium varied among different age groups (Fig 1B and 1C); and the cell densities in all age-groups were reduced (Fig 1B and 1C). In WT stroma, highly organized extracellular matrixes and nuclei stacked in an orthogonal pattern were detected (Fig 1B). In contrast, the cells in the mutant stroma were also disoriented (Fig 1B and 1D), a similar deficit as that of mutant epithelium. Different from that of the mutant epithelium, the mutant stroma became thicker in P30 or older mice, and cell densities in all age-groups were increased (Fig 1B, 1D and 1E). In addition to epithelium and stroma, the mutant endothelium was also disturbed. Whereas WT endothelium exhibited single layer of cells distributed in parallel with the stroma cells (Fig 1B), the mutant endothelial cells were enlarged in size and disoriented in their distribution, but without an obvious change in their cell density (Fig 1B and 1F). Interestingly, excrescences in the endothelial membrane was notable in the P90 mutant cornea (Fig 1B). These morphological alterations (e.g., enlarged cell size, disorganized cell distribution, altered cell density, increased thick-ness, and excrescences in the endothelial membrane) in Vps35^+/-^ cornea resembled in certain degree to that of corneal dystrophy-like deficit.

**Fig 2:**
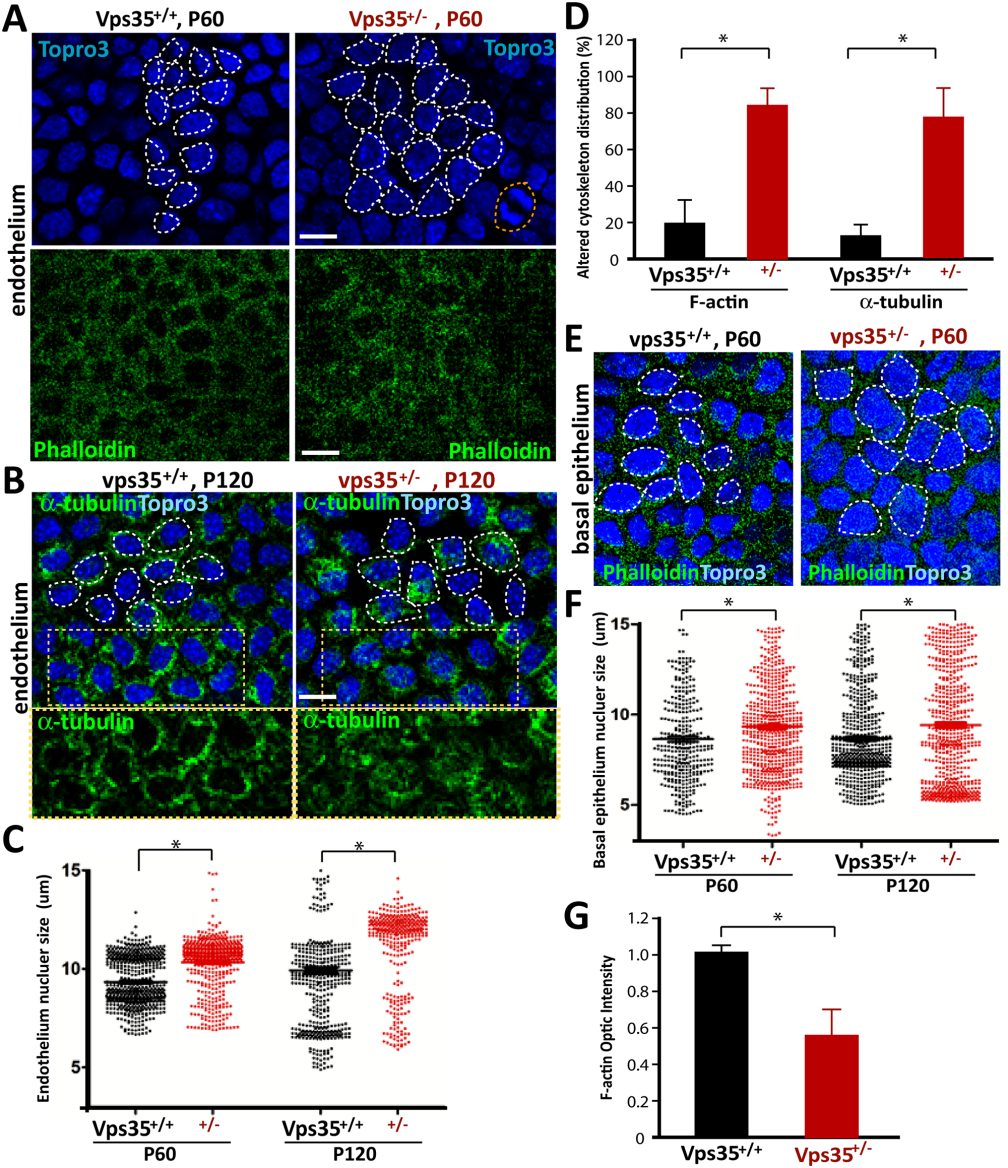
Enlarged nuclei sizes and disorganized cytoskeleton in Vps35^+/-^ corneal epithelium and endothelium. **(A-B)** Immunostaining analysis using anti α-tubulin antibodies or phalloidin (to label F-actin) and Topro3 (to mark nuclei) of flat-mounted corneal endothelium from Vps35^+/+^ and ^+/-^ mice at indicated ages. Representative images were shown. **(C-D)** Quantification analysis of data from **(A-B)**. The nuclei sizes were enlarged and the cytoskeleton structures were disorganized in Vps35^+/-^ corneal endothelium. Cells with cytoskeletal filaments in the nucleus are considered as cells with “altered cytoskeletal distribution” in (D). **(E)** Imaging analysis of phalloidin and Topro3 stained flat-mounted corneal basal epithelium from P60 Vps35^+/+^ and ^+/-^ mice. **(F-G)** Quantification analysis of data from **(E)**. The nuclei sizes were also enlarged and the F-actin filaments were decreased in Vps35^+/-^ corneal basal epithelium. In Fig 2C and 2F, Total 200 nucleus from 4 different mice in each group were measured. Scale bars, 10 μm.

We then verified some of these morphological alterations by fluorescence staining analysis. Indeed, the cell nuclei sizes labeled by Topro3 in mutant cornea, including epithelial, endothelial, and stromal cells, were all enlarged with a disorganized distribution pattern (Fig 2A–2F). Additionally, the phalloidin labeled F-actin filaments and α-tubulin marked microtubules, both critical cytoskeletons that determine cell morphology, appeared to be disorganized in the mutant endothelial cells (Fig 2A, 2B and 2D). F-actin filaments in the mutant basal epithelium were reduced (Fig 2E and 2G). These results are in line with the view of the enlarged nuclei sizes in both epithelial and endothelial cells and disorganized endothelial cytoskeleton/morphology.

### Vps35 expression in cornea

**Fig 3:**
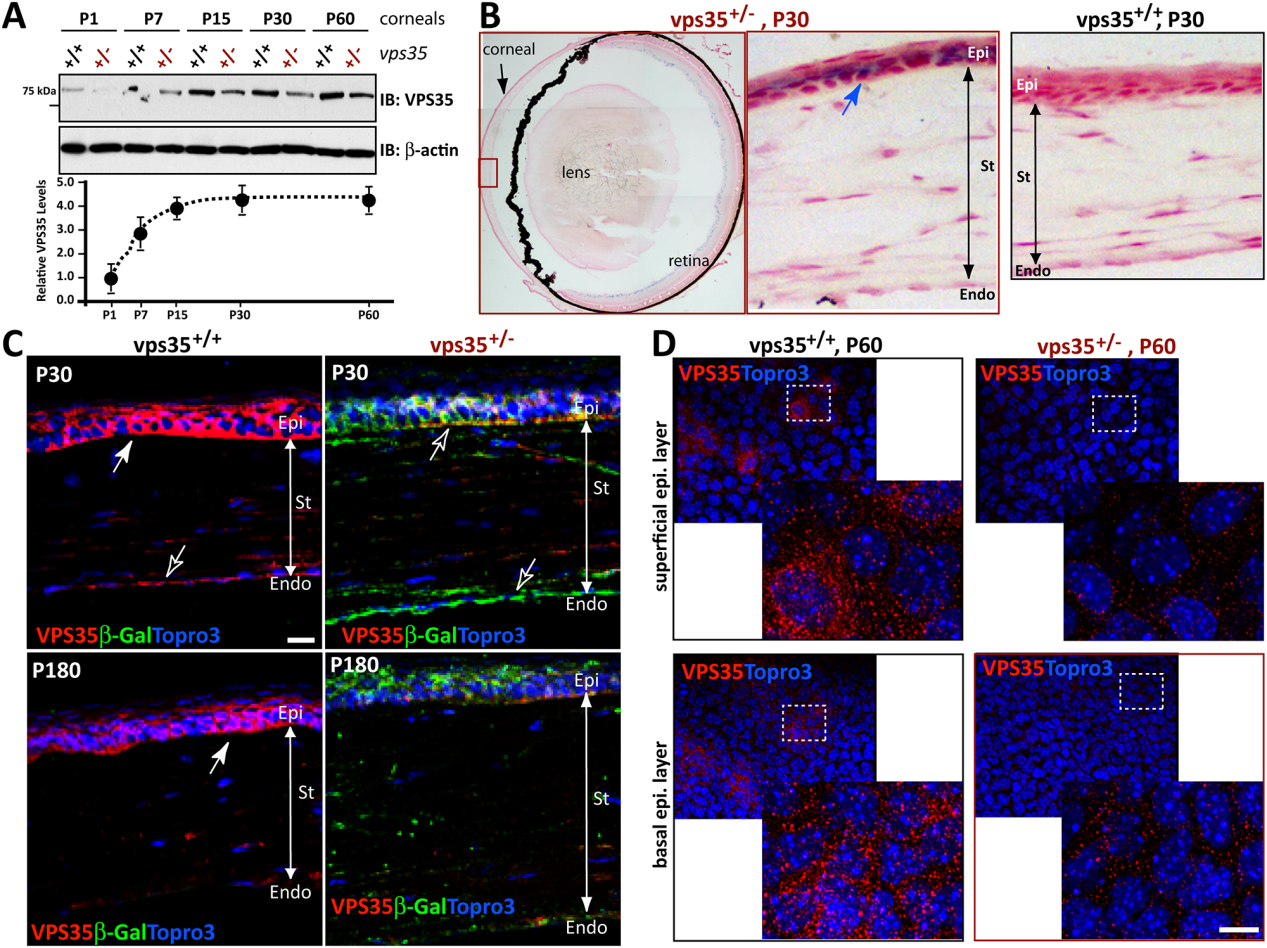
Vps35 expression in developing mouse cornea. **(A)** Western blot analysis of Vps35 expression in cornea from Vps35^+/+^ and ^+/-^ at indicated ages. **(B)** X-gal staining (Blue color) analysis for the LacZ gene expression in Vps35^+/-^ retina and cornea. It was negative in Vps35^+/+^ controls due to lacking LacZ gene insertion. **(C)** Co-immunostaining analysis in cross sections of cornea from P30 and P180 Vps35^+/+^ and ^+/-^ mice using anti-Vps35 andβ-gal antibodies. Representative projected images were shown. **(D)** Confocal imaging analysis of P60 flat-mounted cornea immunostained with anti-Vps35 antibodies. Vps35 was positive in both corneal basal and superficial layers of epithelium. Epi, corneal epithelium; St, corneal stroma; Endo, corneal endothelium. Scale bars, 20 μm.

To understand how Vps35 regulates corneal morphology, we examined Vps35’s expression in mouse cornea. Western blot analysis showed its expression from P1 to all the ages examined, with a marked increase after eyelid opening (~P15) (Fig 3A). An age-dependent increase of Vps35’s expression was also detected in human corneal endothelium microarray database (S2 Fig) [44]. We then took advantage of Vps35^+/-^ mice, in which LacZ gene is knocked in the intron of Vps35 gene [13, 21, 25], thus it was used as a reporter for Vps35’s expression in mouse cornea (Fig 3B and S3 Fig). As the results by Western blot analysis, the LacZ activity (β-gal) was also detected in developing mouse cornea in all the ages examined (Fig 3B and 3C, S3 Fig). Interestingly, the β-gal was distributed in the epithelial layer of Vps35^+/-^, but not Vps35^+/+^, cornea (Fig 3B), demonstrating the specificity of the staining. Vps35 protein distribution in epithelial layer was further confirmed by immunostaining analysis using antibodies against Vps35 and β-gal (Fig 3C and 3D). The immunostaining signal appeared to be specific, as anti-Vps35 signal was reduced, but anti-β-gal was detected, in Vps35^+/-^ cornea, compared to Vps35^+/+^ controls (Fig 3C and 3D). In addition to basal epithelial cells, Vps35 by both anti-Vps35 and β-gal antibodies was detected in stroma cells (Fig 3C), as well as endothelial cells (Fig 3C, indicated by open arrows) in neonatal and young adult cornea. These results suggest that Vps35 is widely expressed in developing mouse cornea, with a peak level at P15.

### Reduced cell proliferation in Vps35-deficient corneal epithelium

**Fig 4:**
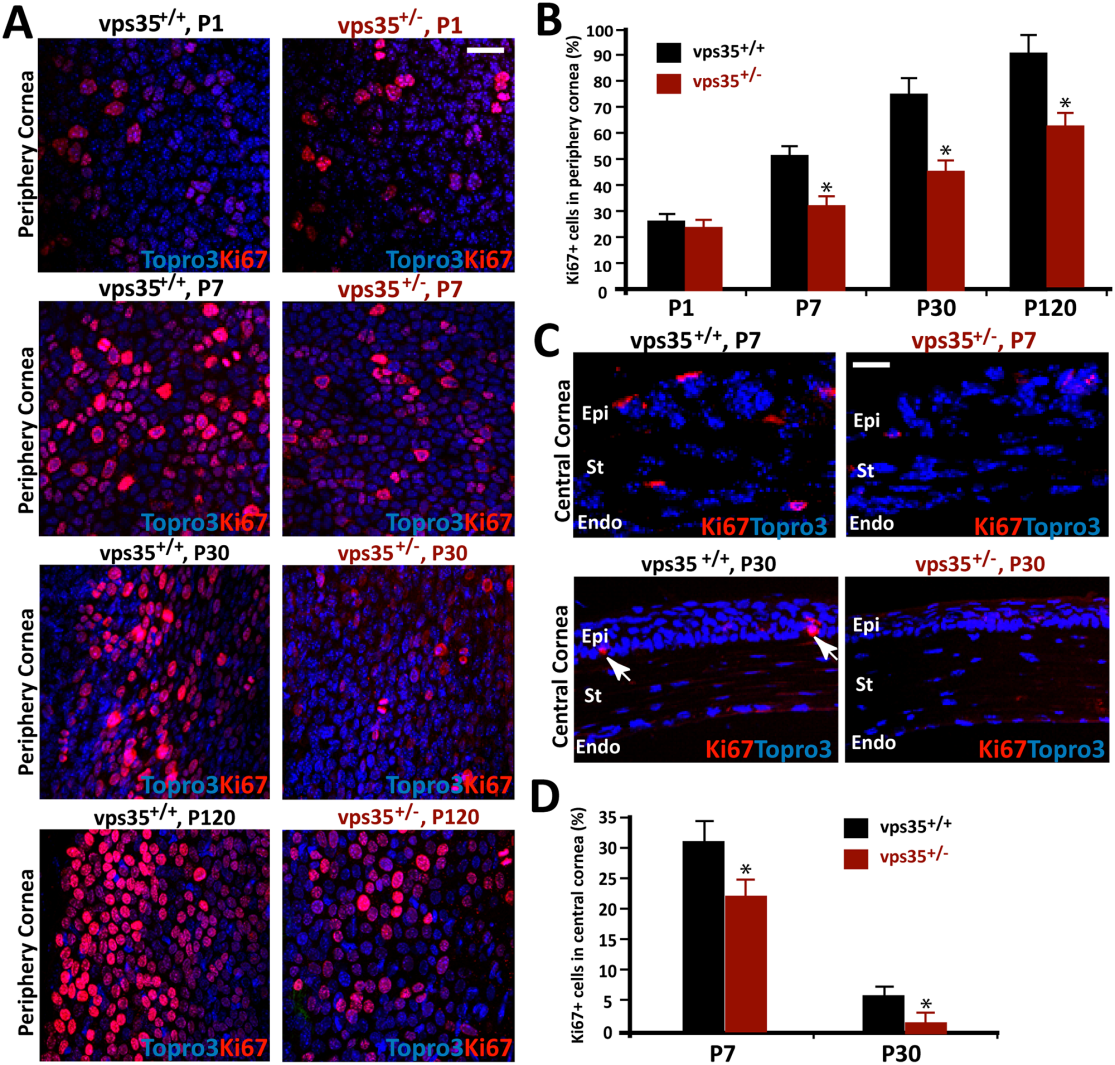
Reduced epithelial cell proliferation in Vps35^+/-^ cornea. **(A-B)** Immunostaining analysis of flat-mounted cornea from Vps35^+/+^ and ^+/-^ mice at indicated ages using anti-Ki67 antibodies. Representative images were shown in (A), and Quantification analysis was presented in (B). At periphery area of cornea (where limbus stem cells located), the Ki67 positive cells were obviously reduced in Vps35^+/-^ cornea, compared with the controls. **(C-D)** Immunostaining analysis of cross sections of cornea from P7 and P30 Vps35^+/+^ and ^+/-^ mice using anti-Ki67 antibodies. Representative images were shown in (C), and Quantification analysis (mean +/- SEM, n = 3 each group of each genotype, *, P < 0.05) was presented in (D). At central area of cornea, the Ki67 positive cells were also decreased in Vps35^+/-^ cornea. Epi, corneal epithelium; St, corneal stroma; Endo, corneal endothelium. Scale bars, 20 μm.

Corneal basal epithelial cells are critical for epithelial cell proliferation, differentiation, and regeneration. The high level of Vps35 expression in this layer and the reduced epithelial cell density in Vps35^+/-^ cornea led to the speculation for Vps35’s function in regulating epithelial cell proliferation. To test this speculation, immunostaining analysis of Ki67, which markers proliferative cells, was carried out in varies aged flat-mounted Vps35^+/+^ and ^+/-^ cornea (Fig 4A and 4B). Indeed, obvious reductions in the number of Ki67 positive cells were detected in the P7 or older mutant cornea (Fig 4A and 4B). Consistently, anti-Ki67 staining analysis of cross sections of P7 and P30 cornea also showed decreased Ki67 positive cells in central regions of Vps35^+/-^ cornea (Fig 4C and 4D). Note that the Ki67 positive cells were detectable not only in P7 epithelial cells, but also in P7 stroma and endothelial cells (Fig 4C), whereas in P30 cornea, Ki67 positive cells were restricted to the basal epithelial cell layer (Fig 4C). Together, these results suggest that Vps35 is necessary for developing corneal cell proliferation, in particularly, the basal epithelial cell proliferation.

### Impaired cell surface targeting of SLC4A11 in Vps35-deficient cells and reduced SLC4A11 in Vps35-deficient corneal endothelium

**Fig 5:**
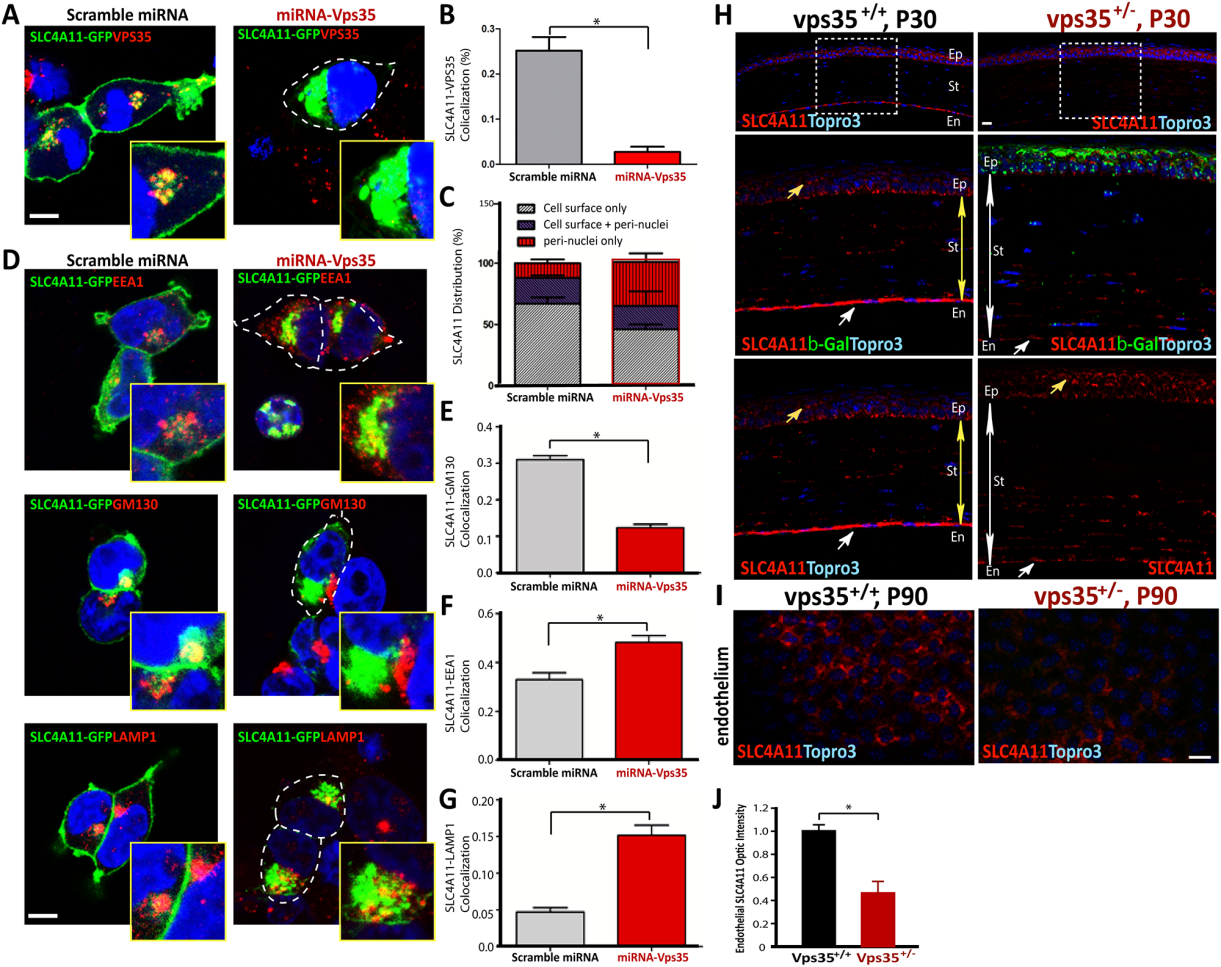
Impaired cell surface targeting of SLC4A11 in Vps35-deficient cells and reduced SLC4A11 in Vps35-deficient corneal endothelium. **(A-G)** HEK293 cells transfected with SLC4A11-GFP with control and miRNA-Vps35 were subjected to co-immunstaining analyses using indicated antibodies. Representative confocal images were shown in (A and D); and quantification analyses (mean +/- SEM, n = 20 cells, *, p<0.05) were presented in (B-C, E-G). Scale bars, 20 μm. **(H)** Co-Immunostaining analysis using anti-SLC4A11 and β-gal antibodies of cross-mounted cornea from P30 Vps35^+/+^ and ^+/-^ mice. Scale bars, 20 μm. **(I-J)** Immunostaining analysis using anti-SLC4A11 antibodies of flat-mounted cornea from P30 and P90 Vps35^+/+^ and ^+/-^ mice. Scale bars, 20 μm. Epi, corneal epithelium; St, corneal stroma; Endo, corneal endothelium. Representative images were shown in (I), and Quantification analysis was presented in (J) (mean +/- SEM, n = 4 mice/each group, *, p<0.05).

The multiple cellular deficits in the mutant cornea implicate a complex of molecular cellular mechanisms that may underlie Vps35 regulation of corneal morphology. As the deficit similar to the pathology of corneal dystrophy, we speculate that Vps35/retromer, via its various cargos in cornea, plays an important role in suppressing corneal stroma edema and promoting cell proliferation. We thus searched the literature for membrane proteins (potential retromer cargos) that are mutated or lost in patients with corneal dystrophy. Among which, SLC4A11 attracted our attention for the following reasons. First, SLC4A11 is a multiple transmembrane domain containing protein that acts as a transporter critical for ion and water homeostasis [45]. Second, numerous mutations in SLC4A11 gene have been identified in multiple corneal dystrophy disorders, including FECD, CHED2, and CDPD [35–37, 39, 40, 46]. Third, a SLC4A11 related family member, SLC4A7, has been identified as a potential cargo of retromer in Hela cells [47]. To test if SLC4A11 is a Vps35’s cargo, we examined whether they form a complex and if Vps35-deficiency results in a defect of SLC4A11’s cellular distribution. HEK293 cells were co-transfected SLC4A11-GFP with control (scramble) and miRNA-Vps35. In control cells, whereas SLC4A11 was mainly distributed in the cell surface, factions of SLC4A11 was indeed co-localized with endogenous Vps35 in the peri-nuclei region (Fig 5A and 5B). Vps35-deficiency by its miRNA resulted in an altered distribution of SLC4A11, and many (~50%) Vps35-deficient cells show impaired SLC4A11’s cell surface distribution (Fig 5A and 5C). Although SLC4A11 was largely distributed in the peri-nuclei regions of Vps35 deficient cells, it’s co-localization with trans-Golgi network was reduced (Fig 5D and 5E), but its distribution in endosomal compartments, both EEA1 labeled early endosomes and Lamp1 marked late endosomes/early lysosomes, was increased (Fig 5D–5G). These results suggest that SLC4A11 appears to be a Vps35/retromer cargo, whose cell surface targeting depends on Vps35’s function.

We further tested this view in WT and Vps35 deficient cornea. As reported, SLC4A11 is largely distributed in the corneal endothelium, but weakly in the basal epithelial cells and stromal cells by immunostaining analysis of cross corneal sections (Fig 5H). In the mutant cross corneal sections, SLC4A11’s endothelial distribution was marked reduced (Fig 5H). We further verified the altered SLC4A11’s distribution by immunostaining analysis of the whole amounts of cornea from P30 and P90 WT and mutant mice. Indeed, the endothelial SLC4A11 was lower, in the mutant cornea than those in WT controls (Fig 5I and 5J). These results support the view for SLC4A11 as a retromer cargo in cornea, implicating a differential retromer regulation of SLC4A11 in a cell type dependent manner.

## Discussion

**Fig 6:**
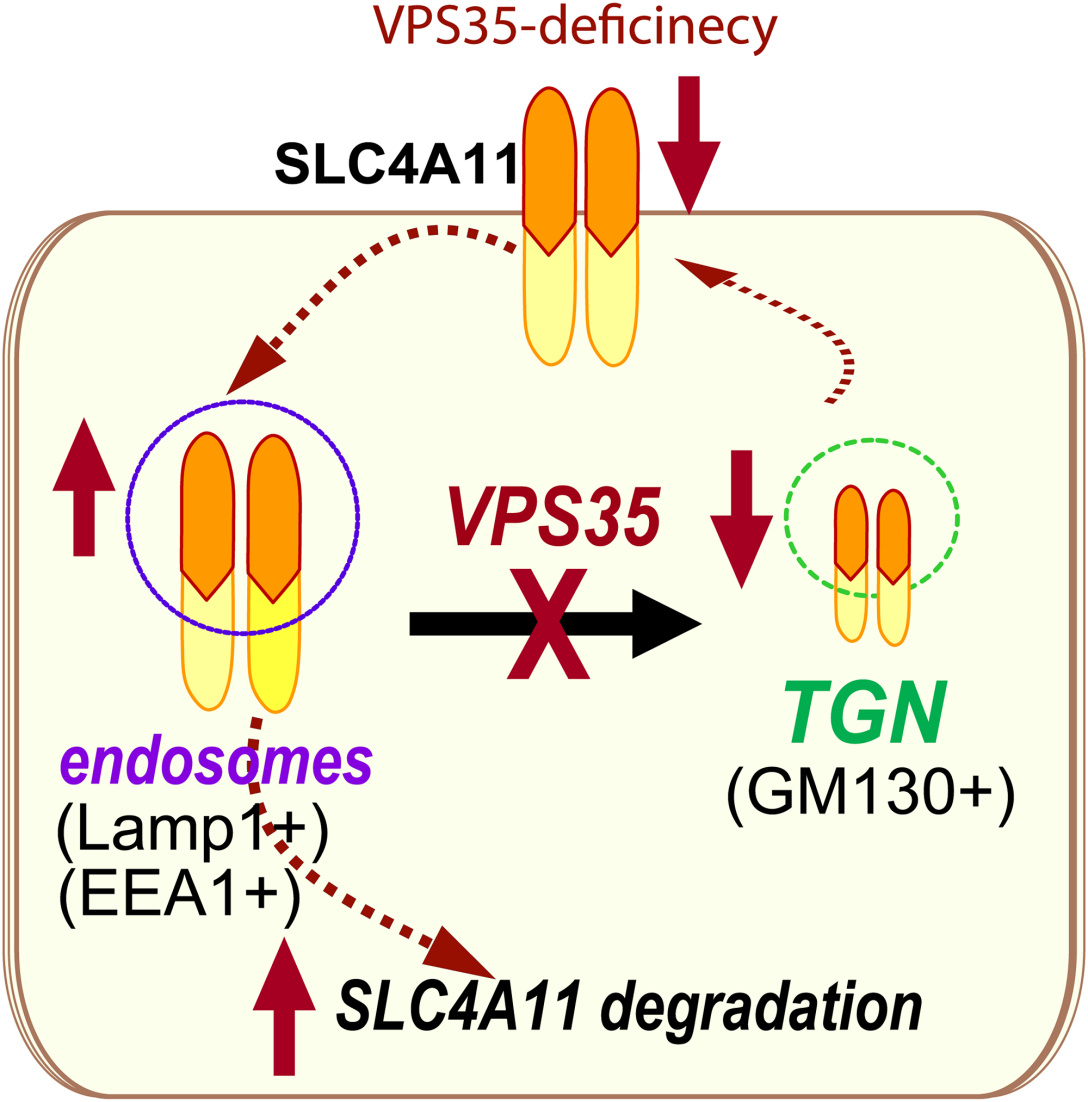
Reduced SLC4A11 co-localization with Vps35/retromer and SLC4A11 cell surface targeting by corneal dystrophy-linked SLC4A11 mutations. Illustration of a working hypothesis for Vps35 regulation of SLC4A11, which may underlie Vps35’s involvement of corneal dystrophy.

Fuchs endothelial corneal dystrophy (FECD) is a common disease characterized by progressive loss of endothelial cells, thickening of the Descemet membrane, and deposition of extracellular matrix in the form of guttae. Advanced cases result in corneal edema and vision loss. Genetic mutations are believed to be a major risk factor for the pathogenesis of FECD, and mutations in several genes, including SLC4A11, have been identified in patients with FECD. However, exactly how these mutations cause the disorder remains to be explored. Here we present evidence for Vps35-deficiency to cause FECD-like pathology in a mouse model. First, Vps35 is expressed in developing mouse cornea; Second, Vps35-deficiency cornea show dystrophy like morphology, with increased cornea thickness and cell size, disorganized cell distribution, excrescences in the Descemet membrane and corneal edema. Third, cell proliferation is reduced in Vps35-defcient cornea. Finally, the endothelial SLC4A11 protein level was lower, in the Vps35-defcient cornea than those in WT controls. Based on our cell biological studies, we have proposed a working model depicted in Fig 6. In this model, corneal endothelial Vps35 is necessary for SLC4A11’s endosome-to-Golgi trafficking, and thus promoting SLC4A11’s cell surface targeting and protein stability, which may underlie Vps35’s involvement in the pathogenesis of corneal dystrophy.

How does loss of Vps35 cause corneal dystrophy? Vps35, a key component of retromer, selectively regulates endosome-to-Golgi retrieval of membrane proteins. It is possible for several Vps35/retromer’s cargos in corneal endothelium to underlie Vps35’s function. However, we speculate that SLC4A11 acts as a new Vps35 cargo in this event for the following reasons. First, our cellular and in vivo studies have shown that Vps35 is necessary for SLC4A11 cell surface targeting (Fig 5), and Vps35 deficiency caused a loss of SLC4A11 protein (Fig 5); Second, mutations in SLC4A11gene, which result in a loss of SLC4A11’s function [40, 45], have been identified in patients with three genetic corneal dystrophies, including CHED2, Harboyan syndrome, and FECD. Third, SLC4A11 knock out mouse models show the height of the corneal basal epithelial cells and the basal epithelial cell/total corneal thickness are significantly increased [45, 48], similar to that of Vps35-deficient mice. Finally, SLC4A11 is critical to prevent fluid accumulation in cornea; and the selective transmembrane water conductance by SLC4A11 could control cell size and affect cell division/proliferation [43, 45, 49, 50]. These observations thus support the view for SLC4A11 as a Vps35 cargo in regulation corneal dystrophy. However, it remains to be determined if Vps35 mutation or deficiency occurs in corneal dystrophy patients, and if expression of SLC4A11 could attenuate corneal dystrophy in Vps35 mutant mice.

As we mentioned, SLC4A7, another member of the SLC4 gene family is known to be a retromer cargo in Hela cells by unbiased screen [47]. Interestingly, mice lacking SLC4A7 develop blindness and auditory impairment due to the degeneration of sensory receptors in the eye and inner ear [27]. Meanwhile, mutations in SLC4A4, a traditional sodium bicarbonate cotransporters, cause proximal renal tubular acidosis as well as ocular anomalies, such as glaucoma, cataracts, and band keratopathy in human [41, 51]. Both SLC4A7 and SLC4A4 are expressed in murine corneal endothelial cells, but their expression level are relative lower than SLC4A11 [52]. Recent study establishes expression profiles for each SLC4 family member in primary corneal endothelium, and the SLC4A11 has the highest expression in corneal endothelial [52], indicating that it plays a pivotal role in transporting solutes in the corneal endothelium, which may be important for preventing corneal dystrophy. Nevertheless, our study does not exclude the possibility that other cargo proteins may be negatively impacted by Vps35 haploinsufficiency and involved in the pathogenesis of corneal dystrophy.

## Supporting information

S1 Fig Normal lens morphology in Vps35^+/-^ mice. Immunostaining analysis using anti-β-catenin antibodies of cross-sectioned lens from P60 Vps35^+/+^ and ^+/-^ mice. Scale bars, 20 μm.
(TIF)

S2 Fig Vps35’s mRNA expression in human cornea. Vps35 mRNA levels in human corneal endothelium from pediatric (4–11 years old) and adult (53–70 years old) donor corneas as determined by gene expression microarray data set (GEO GDS5432).
(TIF)

S3 Fig X-gal activity in P15 and P90 Vps35^+/-^ cornea. X-gal staining (Blue color, single arrow) showed *lacZ gene* expression in the corneal epithelium of Vps35^+/-^ mice at P15 and P90. Epi, corneal epithelium; St, corneal stroma; Endo, corneal endothelium. Scale bar, 50 μm.
(TIF)

## References

[1] Seaman MN, Marcusson EG, Cereghino JL, Emr SD. Endosome to Golgi retrieval of the vacuolar protein sorting receptor, Vps10p, requires the function of the VPS29, VPS30, and VPS35 gene products. J Cell Biol. 1997;137(1):79–92. Epub 1997/04/07. 9105038 10.1083/jcb.137.1.79 PMC2139870

[2] Bonifacino JS, Hurley JH. Retromer. Curr Opin Cell Biol. 2008;20(4):427–36. Epub 2008/05/13. doi: 10.1016/j.ceb.2008.03.009 18472259 10.1016/j.ceb.2008.03.009 PMC2833274

[3] McGough IJ, Cullen PJ. Recent advances in retromer biology. Traffic. 2011;12(8):963–71. Epub 2011/04/06. doi: 10.1111/j.1600-0854.2011.01201.x . 21463457 10.1111/j.1600-0854.2011.01201.x

[4] Burd C, Cullen PJ. Retromer: a master conductor of endosome sorting. Cold Spring Harbor perspectives in biology. 2014;6(2). doi: 10.1101/cshperspect.a016774 24492709 10.1101/cshperspect.a016774 PMC3941235

[5] Cullen PJ, Korswagen HC. Sorting nexins provide diversity for retromer-dependent trafficking events. Nature cell biology. 2011;14(1):29–37. doi: 10.1038/ncb2374 22193161 10.1038/ncb2374 PMC3613977

[6] Seaman MN. Recycle your receptors with retromer. Trends Cell Biol. 2005;15(2):68–75. doi: 10.1016/j.tcb.2004.12.004 . 15695093 10.1016/j.tcb.2004.12.004

[7] Gallon M, Cullen PJ. Retromer and sorting nexins in endosomal sorting. Biochemical Society transactions. 2015;43(1):33–47. doi: 10.1042/BST20140290 . 25619244 10.1042/BST20140290

[8] Small SA, Petsko GA. Retromer in Alzheimer disease, Parkinson disease and other neurological disorders. Nature reviews Neuroscience. 2015;16(3):126–32. doi: 10.1038/nrn3896 . 25669742 10.1038/nrn3896

[9] Vilarino-Guell C, Wider C, Ross OA, Dachsel JC, Kachergus JM, Lincoln SJ, et al VPS35 mutations in Parkinson disease. American journal of human genetics. 2011;89(1):162–7. doi: 10.1016/j.ajhg.2011.06.001 21763482 10.1016/j.ajhg.2011.06.001 PMC3135796

[10] Zimprich A, Benet-Pages A, Struhal W, Graf E, Eck SH, Offman MN, et al A mutation in VPS35, encoding a subunit of the retromer complex, causes late-onset Parkinson disease. American journal of human genetics. 2011;89(1):168–75. Epub 2011/07/19. doi: 10.1016/j.ajhg.2011.06.008 21763483 10.1016/j.ajhg.2011.06.008 PMC3135812

[11] Rovelet-Lecrux A, Charbonnier C, Wallon D, Nicolas G, Seaman MN, Pottier C, et al De novo deleterious genetic variations target a biological network centered on Abeta peptide in early-onset Alzheimer disease. Molecular psychiatry. 2015;20(9):1046–56. doi: 10.1038/mp.2015.100 . 26194182 10.1038/mp.2015.100

[12] Small SA, Kent K, Pierce A, Leung C, Kang MS, Okada H, et al Model-guided microarray implicates the retromer complex in Alzheimer's disease. Ann Neurol. 2005;58(6):909–19. Epub 2005/11/30. doi: 10.1002/ana.20667 . 16315276 10.1002/ana.20667

[13] Wen L, Tang FL, Hong Y, Luo SW, Wang CL, He W, et al VPS35 haploinsufficiency increases Alzheimer's disease neuropathology. J Cell Biol. 2011;195(5):765–79. Epub 2011/11/23. doi: 10.1083/jcb.201105109 22105352 10.1083/jcb.201105109 PMC3257571

[14] Miura E, Hasegawa T, Konno M, Suzuki M, Sugeno N, Fujikake N, et al VPS35 dysfunction impairs lysosomal degradation of alpha-synuclein and exacerbates neurotoxicity in a Drosophila model of Parkinson's disease. Neurobiology of disease. 2014;71:1–13. doi: 10.1016/j.nbd.2014.07.014 . 25107340 10.1016/j.nbd.2014.07.014

[15] Tsika E, Glauser L, Moser R, Fiser A, Daniel G, Sheerin UM, et al Parkinson's disease-linked mutations in VPS35 induce dopaminergic neurodegeneration. Human molecular genetics. 2014;23(17):4621–38. doi: 10.1093/hmg/ddu178 24740878 10.1093/hmg/ddu178 PMC4119414

[16] Dhungel N, Eleuteri S, Li LB, Kramer NJ, Chartron JW, Spencer B, et al Parkinson's disease genes VPS35 and EIF4G1 interact genetically and converge on alpha-synuclein. Neuron. 2015;85(1):76–87. doi: 10.1016/j.neuron.2014.11.027 25533483 10.1016/j.neuron.2014.11.027 PMC4289081

[17] Malik BR, Godena VK, Whitworth AJ. VPS35 pathogenic mutations confer no dominant toxicity but partial loss of function in Drosophila and genetically interact with parkin. Human molecular genetics. 2015;24(21):6106–17. Epub 2015/08/08. doi: 10.1093/hmg/ddv322 26251041 10.1093/hmg/ddv322 PMC4599670

[18] Wang W, Wang X, Fujioka H, Hoppel C, Whone AL, Caldwell MA, et al Parkinson's disease-associated mutant VPS35 causes mitochondrial dysfunction by recycling DLP1 complexes. Nature medicine. 2016;22(1):54–63. doi: 10.1038/nm.3983 26618722 10.1038/nm.3983 PMC4826611

[19] Tang FL, Erion JR, Tian Y, Liu W, Yin DM, Ye J, et al VPS35 in Dopamine Neurons Is Required for Endosome-to-Golgi Retrieval of Lamp2a, a Receptor of Chaperone-Mediated Autophagy That Is Critical for alpha-Synuclein Degradation and Prevention of Pathogenesis of Parkinson's Disease. The Journal of neuroscience: the official journal of the Society for Neuroscience. 2015;35(29):10613–28. doi: 10.1523/JNEUROSCI.0042-15.2015 26203154 10.1523/JNEUROSCI.0042-15.2015 PMC4510296

[20] Tang FL, Liu W, Hu JX, Erion JR, Ye J, Mei L, et al VPS35 Deficiency or Mutation Causes Dopaminergic Neuronal Loss by Impairing Mitochondrial Fusion and Function. Cell reports. 2015;12(10):1631–43. doi: 10.1016/j.celrep.2015.08.001 26321632 10.1016/j.celrep.2015.08.001 PMC4565770

[21] Wang CL, Tang FL, Peng Y, Shen CY, Mei L, Xiong WC. VPS35 regulates developing mouse hippocampal neuronal morphogenesis by promoting retrograde trafficking of BACE1. Biol Open. 2012;1(12):1248–57. Epub 2012/12/22. doi: 10.1242/bio.20122451 23259059 10.1242/bio.20122451 PMC3522886

[22] Liu W, Tang FL, Erion J, Xiao H, Ye J, Xiong WC. Vps35 haploinsufficiency results in degenerative-like deficit in mouse retinal ganglion neurons and impairment of optic nerve injury-induced gliosis. Molecular brain. 2014;7:10 doi: 10.1186/1756-6606-7-10 24512632 10.1186/1756-6606-7-10 PMC4016418

[23] Pocha SM, Wassmer T, Niehage C, Hoflack B, Knust E. Retromer controls epithelial cell polarity by trafficking the apical determinant Crumbs. Current biology: CB. 2011;21(13):1111–7. doi: 10.1016/j.cub.2011.05.007 . 21700461 10.1016/j.cub.2011.05.007

[24] de Groot RE, Farin HF, Macurkova M, van Es JH, Clevers HC, Korswagen HC. Retromer dependent recycling of the Wnt secretion factor Wls is dispensable for stem cell maintenance in the mammalian intestinal epithelium. PloS one. 2013;8(10):e76971 doi: 10.1371/journal.pone.0076971 24130821 10.1371/journal.pone.0076971 PMC3793972

[25] Xia WF, Tang FL, Xiong L, Xiong S, Jung JU, Lee DH, et al Vps35 loss promotes hyperresorptive osteoclastogenesis and osteoporosis via sustained RANKL signaling. J Cell Biol. 2013;200(6):821–37. Epub 2013/03/20. doi: 10.1083/jcb.201207154 23509071 10.1083/jcb.201207154 PMC3601351

[26] Xiong L, Xia WF, Tang FL, Pan JX, Mei L, Xiong WC. Retromer in Osteoblasts Interacts With Protein Phosphatase 1 Regulator Subunit 14C, Terminates Parathyroid Hormone's Signaling, and Promotes Its Catabolic Response. EBioMedicine. 2016;9:45–60. doi: 10.1016/j.ebiom.2016.05.028 27333042 10.1016/j.ebiom.2016.05.028 PMC4972523

[27] Tian Y, Tang FL, Sun X, Wen L, Mei L, Tang BS, et al VPS35-deficiency results in an impaired AMPA receptor trafficking and decreased dendritic spine maturation. Molecular brain. 2015;8(1):70 doi: 10.1186/s13041-015-0156-4 26521016 10.1186/s13041-015-0156-4 PMC4628247

[28] Eghrari AO, Riazuddin SA, Gottsch JD. Overview of the Cornea: Structure, Function, and Development. Progress in molecular biology and translational science. 2015;134:7–23. doi: 10.1016/bs.pmbts.2015.04.001 . 26310146 10.1016/bs.pmbts.2015.04.001

[29] Zieske JD. Corneal development associated with eyelid opening. The International journal of developmental biology. 2004;48(8–9):903–11. doi: 10.1387/ijdb.041860jz . 15558481 10.1387/ijdb.041860jz

[30] Kim EK, Lee H, Choi SI. Molecular Pathogenesis of Corneal Dystrophies: Schnyder Dystrophy and Granular Corneal Dystrophy type 2. Progress in molecular biology and translational science. 2015;134:99–115. doi: 10.1016/bs.pmbts.2015.05.003 . 26310152 10.1016/bs.pmbts.2015.05.003

[31] Schorderet D. Corneal Dystrophies: Overview and Summary. Progress in molecular biology and translational science. 2015;134:73–8. doi: 10.1016/bs.pmbts.2015.04.004 . 26310150 10.1016/bs.pmbts.2015.04.004

[32] Lin ZN, Chen J, Cui HP. Characteristics of corneal dystrophies: a review from clinical, histological and genetic perspectives. International journal of ophthalmology. 2016;9(6):904–13. doi: 10.18240/ijo.2016.06.20 27366696 10.18240/ijo.2016.06.20 PMC4916151

[33] Vedana G, Villarreal G Jr., Jun AS. Fuchs endothelial corneal dystrophy: current perspectives. Clinical ophthalmology. 2016;10:321–30. doi: 10.2147/OPTH.S83467 26937169 10.2147/OPTH.S83467 PMC4762439

[34] Klintworth GK. Corneal dystrophies. Orphanet journal of rare diseases. 2009;4:7 doi: 10.1186/1750-1172-4-7 19236704 10.1186/1750-1172-4-7 PMC2695576

[35] Patel SP, Parker MD. SLC4A11 and the Pathophysiology of Congenital Hereditary Endothelial Dystrophy. BioMed research international. 2015;2015:475392 doi: 10.1155/2015/475392 26451371 10.1155/2015/475392 PMC4588344

[36] Jiao X, Sultana A, Garg P, Ramamurthy B, Vemuganti GK, Gangopadhyay N, et al Autosomal recessive corneal endothelial dystrophy (CHED2) is associated with mutations in SLC4A11. Journal of medical genetics. 2007;44(1):64–8. doi: 10.1136/jmg.2006.044644 16825429 10.1136/jmg.2006.044644 PMC2597914

[37] Desir J, Moya G, Reish O, Van Regemorter N, Deconinck H, David KL, et al Borate transporter SLC4A11 mutations cause both Harboyan syndrome and non-syndromic corneal endothelial dystrophy. Journal of medical genetics. 2007;44(5):322–6. doi: 10.1136/jmg.2006.046904 17220209 10.1136/jmg.2006.046904 PMC2597979

[38] Zhu AY, Eberhart CG, Jun AS. Fuchs endothelial corneal dystrophy: a neurodegenerative disorder? JAMA ophthalmology. 2014;132(4):377–8. doi: 10.1001/jamaophthalmol.2013.7993 24504267 10.1001/jamaophthalmol.2013.7993 PMC4324604

[39] Kodaganur SG, Kapoor S, Veerappa AM, Tontanahal SJ, Sarda A, Yathish S, et al Mutation analysis of the SLC4A11 gene in Indian families with congenital hereditary endothelial dystrophy 2 and a review of the literature. Molecular vision. 2013;19:1694–706. 23922488 PMC3733908

[40] Roy S, Praneetha DC, Vendra VP. Mutations in the Corneal Endothelial Dystrophy-Associated Gene SLC4A11 Render the Cells More Vulnerable to Oxidative Insults. Cornea. 2015;34(6):668–74. doi: 10.1097/ICO.0000000000000421 . 25811729 10.1097/ICO.0000000000000421

[41] Kao L, Azimov R, Shao XM, Frausto RF, Abuladze N, Newman D, et al Multifunctional ion transport properties of human SLC4A11: comparison of the SLC4A11-B and SLC4A11-C variants. American journal of physiology Cell physiology. 2016;311(5):C820–C30. doi: 10.1152/ajpcell.00233.2016 27581649 10.1152/ajpcell.00233.2016 PMC5130583

[42] Loganathan SK, Lukowski CM, Casey JR. The cytoplasmic domain is essential for transport function of the integral membrane transport protein SLC4A11. American journal of physiology Cell physiology. 2016;310(2):C161–74. doi: 10.1152/ajpcell.00246.2015 26582474 10.1152/ajpcell.00246.2015 PMC4719032

[43] Loganathan SK, Schneider HP, Morgan PE, Deitmer JW, Casey JR. Functional assessment of SLC4A11, an integral membrane protein mutated in corneal dystrophies. American journal of physiology Cell physiology. 2016;311(5):C735–C48. doi: 10.1152/ajpcell.00078.2016 . 27558157 10.1152/ajpcell.00078.2016 PMC5130586

[44] Frausto RF, Wang C, Aldave AJ. Transcriptome analysis of the human corneal endothelium. Investigative ophthalmology & visual science. 2014;55(12):7821–30. doi: 10.1167/iovs.14-15021 25377225 10.1167/iovs.14-15021 PMC4258927

[45] Vilas GL, Loganathan SK, Liu J, Riau AK, Young JD, Mehta JS, et al Transmembrane water-flux through SLC4A11: a route defective in genetic corneal diseases. Human molecular genetics. 2013;22(22):4579–90. doi: 10.1093/hmg/ddt307 23813972 10.1093/hmg/ddt307 PMC3889808

[46] Vithana EN, Morgan PE, Ramprasad V, Tan DT, Yong VH, Venkataraman D, et al SLC4A11 mutations in Fuchs endothelial corneal dystrophy. Human molecular genetics. 2008;17(5):656–66. doi: 10.1093/hmg/ddm337 . 18024964 10.1093/hmg/ddm337

[47] Steinberg F, Gallon M, Winfield M, Thomas EC, Bell AJ, Heesom KJ, et al A global analysis of SNX27-retromer assembly and cargo specificity reveals a function in glucose and metal ion transport. Nature cell biology. 2013;15(5):461–71. doi: 10.1038/ncb2721 23563491 10.1038/ncb2721 PMC4052425

[48] Lopez IA, Rosenblatt MI, Kim C, Galbraith GC, Jones SM, Kao L, et al Slc4a11 gene disruption in mice: cellular targets of sensorineuronal abnormalities. The Journal of biological chemistry. 2009;284(39):26882–96. 19586905 10.1074/jbc.M109.008102 PMC2785376

[49] Vilas GL, Loganathan SK, Quon A, Sundaresan P, Vithana EN, Casey J. Oligomerization of SLC4A11 protein and the severity of FECD and CHED2 corneal dystrophies caused by SLC4A11 mutations. Human mutation. 2012;33(2):419–28. 22072594 10.1002/humu.21655

[50] Liu J, Seet LF, Koh LW, Venkatraman A, Venkataraman D, Mohan RR, et al Depletion of SLC4A11 causes cell death by apoptosis in an immortalized human corneal endothelial cell line. Investigative ophthalmology & visual science. 2012;53(7):3270–9. doi: 10.1167/iovs.11-8724 22447871 10.1167/iovs.11-8724 PMC4007482

[51] Dinour D, Chang MH, Satoh J, Smith BL, Angle N, Knecht A, et al A novel missense mutation in the sodium bicarbonate cotransporter (NBCe1/SLC4A4) causes proximal tubular acidosis and glaucoma through ion transport defects. The Journal of biological chemistry. 2004;279(50):52238–46. doi: 10.1074/jbc.M406591200 . 15471865 10.1074/jbc.M406591200

[52] Shei W, Liu J, Htoon HM, Aung T, Vithana EN. Differential expression of the Slc4 bicarbonate transporter family in murine corneal endothelium and cell culture. Molecular vision. 2013;19:1096–106. 23734078 PMC3668641

