# Supplementary figures and images for: Vps35-deficiency impairs SLC4A11 trafficking and promotes corneal dystrophy
Source: PLoS One. 2017 Sep 21;12(9):e0184906. doi: 10.1371/journal.pone.0184906 (PMC5608277; doi:10.1371/journal.pone.0184906)

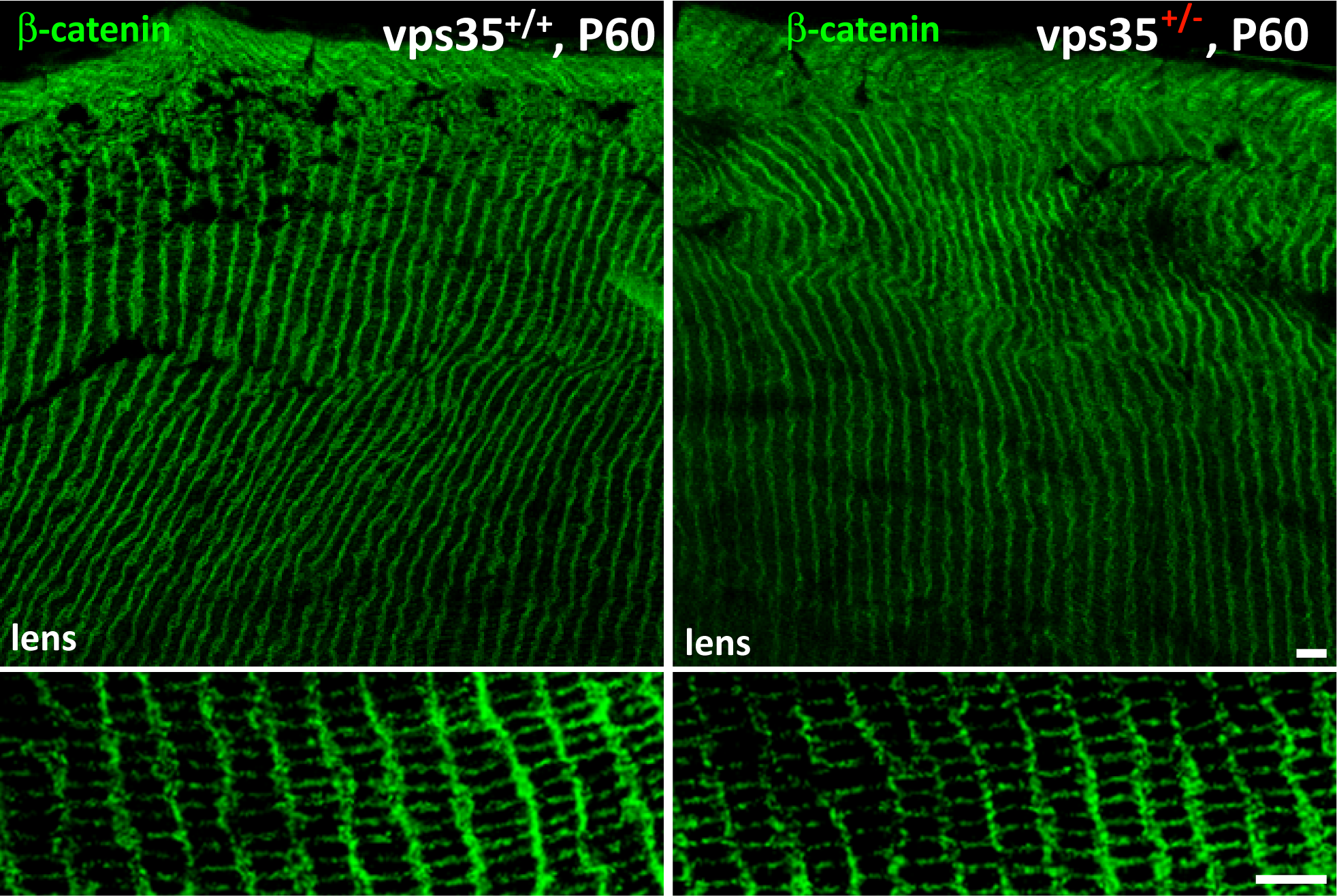

Supplement: S1 Fig — Immunostaining analysis using anti-β-catenin antibodies of cross-sectioned lens from P60 Vps35+/+ and +/- mice. Scale bars, 20 μm. (TIF) [file pone.0184906.s001.tif]

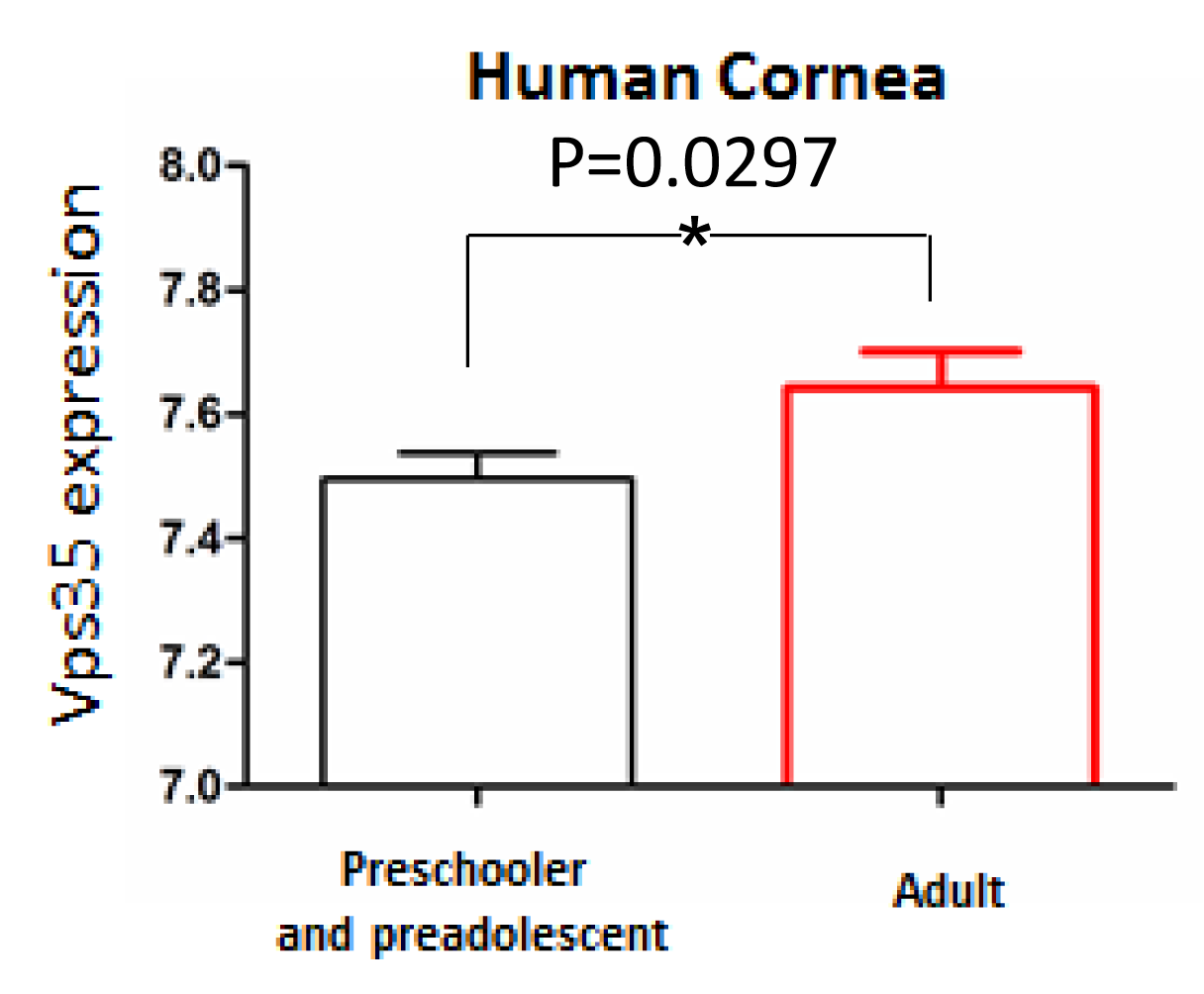

Supplement: S2 Fig — Vps35 mRNA levels in human corneal endothelium from pediatric (4–11 years old) and adult (53–70 years old) donor corneas as determined by gene expression microarray data set (GEO GDS5432). (TIF) [file pone.0184906.s002.tif]

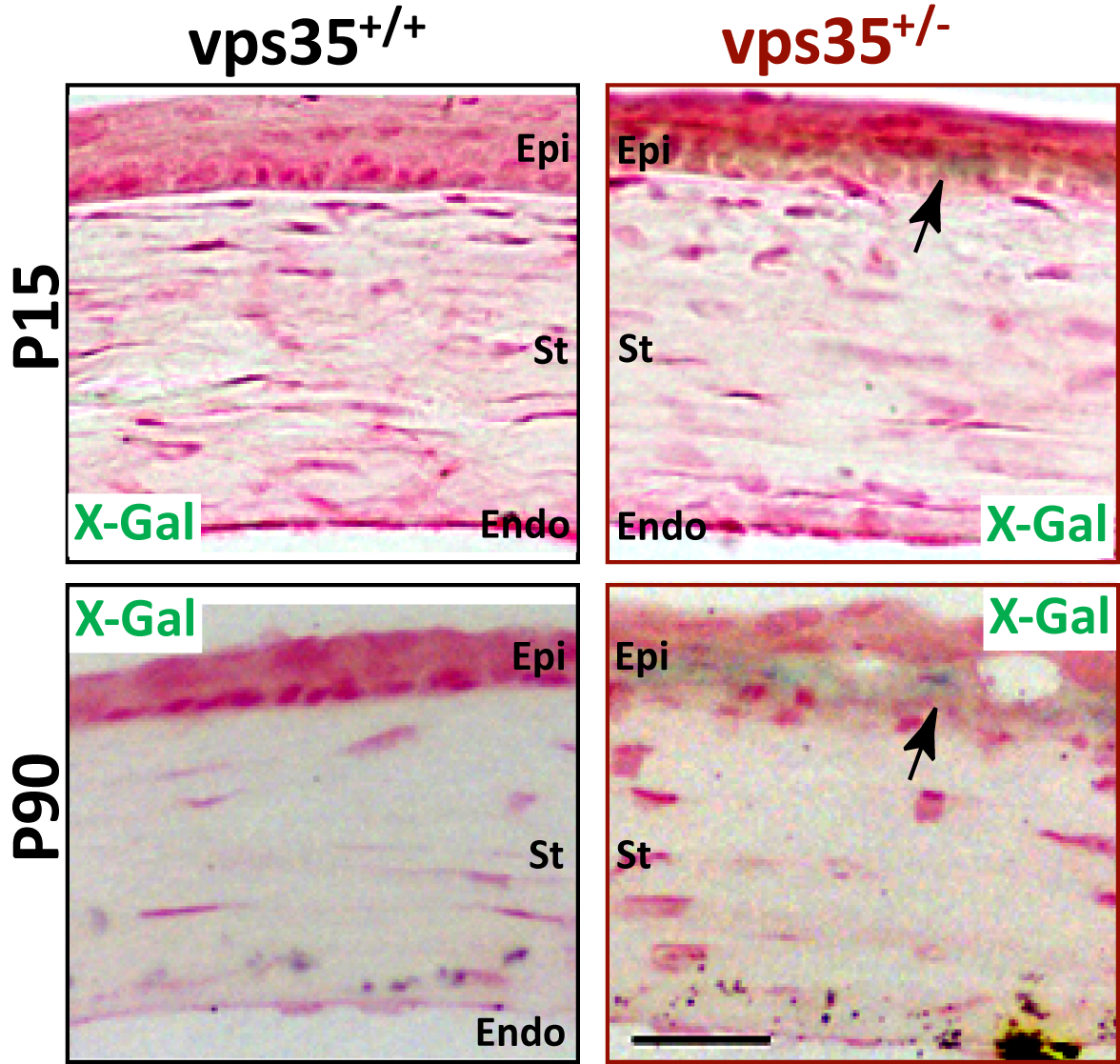

Supplement: S3 Fig — X-gal staining (Blue color, single arrow) showed lacZ gene expression in the corneal epithelium of Vps35+/- mice at P15 and P90. Epi, corneal epithelium; St, corneal stroma; Endo, corneal endothelium. Scale bar, 50 μm. (TIF) [file pone.0184906.s003.tif]
